# Comparison between renal denervation and metoprolol on the susceptibility of ventricular arrhythmias in rats with myocardial infarction

**DOI:** 10.1038/s41598-018-28562-z

**Published:** 2018-07-05

**Authors:** Wanying Jiang, Chu Chen, Junyu Huo, Dasheng Lu, Zhixin Jiang, Jie Geng, Hai Xu, Qijun Shan

**Affiliations:** 1 Department of Cardiology, The First Affiliated Hospital of Nanjing Medical University, Nanjing, 210029 China; 2 Department of Cardiology, The Second Affiliated Hospital of Wannan Medical College, Wuhu, 241000 China

## Abstract

Ventricular arrhythmias (VAs) are the leading cause of sudden cardiac death in patients with myocardial infarction (MI). We sought to compare effects of renal denervation (RDN) and metoprolol on VAs after MI. Fifty-four male Sprague-Dawley rats underwent ligation of left anterior descending coronary artery to induce MI, while 6 rats served as Control. Metoprolol was given 20 mg/kg/day for 5 weeks after MI surgery. RDN/Sham-RDN procedure was performed at 1 week after MI. At 5 weeks after MI, electrical programmed stimulation (EPS) was performed in all groups for evaluation of VAs. After EPS, heart and kidneys were harvested. Compared with MI group, RDN and metoprolol significantly decreased the incidence of VAs, and RDN is superior to metoprolol. Compared with metoprolol group, Masson staining showed that RDN significantly reduced the myocardial fibrosis. Both RDN and metoprolol decreased the protein expression of connexin43 (Cx43) compared with MI group, while only RDN lighted this decrease remarkably. Immunohistochemical staining of Tyrosine hydroxylase (TH) and growth associated protein 43 (GAP43) revealed that RDN and metoprolol had similar effect on reducing densities of sympathetic nerve in infarction border zone. According to this study, RDN is more effective in reducing VAs than metoprolol in ischemic cardiomyopathy model.

## Introduction

According to World Health Organization statistics, Myocardial infarction (MI) is the leading cause of death in human^1^. Arrhythmias, especially ventricular arrhythmias (VAs), are the major causes of sudden death in MI patients. Previous studies showed that many factors involved in the pathogenesis of VAs after MI, including the gap junction remodeling^2^, sympathetic neural remodeling^3^, cardiac fibrosis^4^ as well as electrical remodeling^5^.

Sympathetic nerve remodeling^6,7^ refers to a series of pathophysiological changes after MI, including myocardial denervation, nerve sprouting, sympathetic over-regeneration and high domination, ultimately developing into electrophysiological heterogeneity. This may form the basis for increased susceptibility of VAs in rats with ischemic cardiomyopathy^8^.

Connexin, abundant in normal cardiac tissue, plays an important role in the electrical synchronization of cardiomyocyte contraction. After MI, the expression and distribution of connexin in infarcted myocardial tissue become irregular^9^. Abnormal expression and distribution of connexin results in the gap junction remodeling, which is thought to be an important arrhythmogenic substrate.

Renal denervation (RDN), as a novel and safe method^10^, is mainly used to treat patients with resistant hypertension^11^. Besides, RDN also has a variety of roles such as reducing myocardial fibrosis^12^, promoting angiogenesis after MI^13^, and improving ventricular remodeling in heart failure. Several clinical studies have shown that RDN can reduce the incidence of arrhythmias^14^, including ventricular electrical storm^15^, atrial fibrillation^16^ and other types of arrhythmias^17^. Metoprolol, a classical β-blocker, has a positive effect on improving the long-term survival in patients with MI^18,19^. Many clinical studies showed that metoprolol can reduce the infarcted area^20^, decrease the incidence of recurrent myocardial ischemia^21^ and reduce the risk of malignant arrhythmias^18^. But research on comparison between RDN and metoprolol is insufficient. And the potential mechanistic evaluation remains unclear. In this study, we established MI model to investigate the effect and mechanism of RDN on VAs after MI and compared with metoprolol.

## Results

### Cardiac function at 1 week

**Figure 1:**
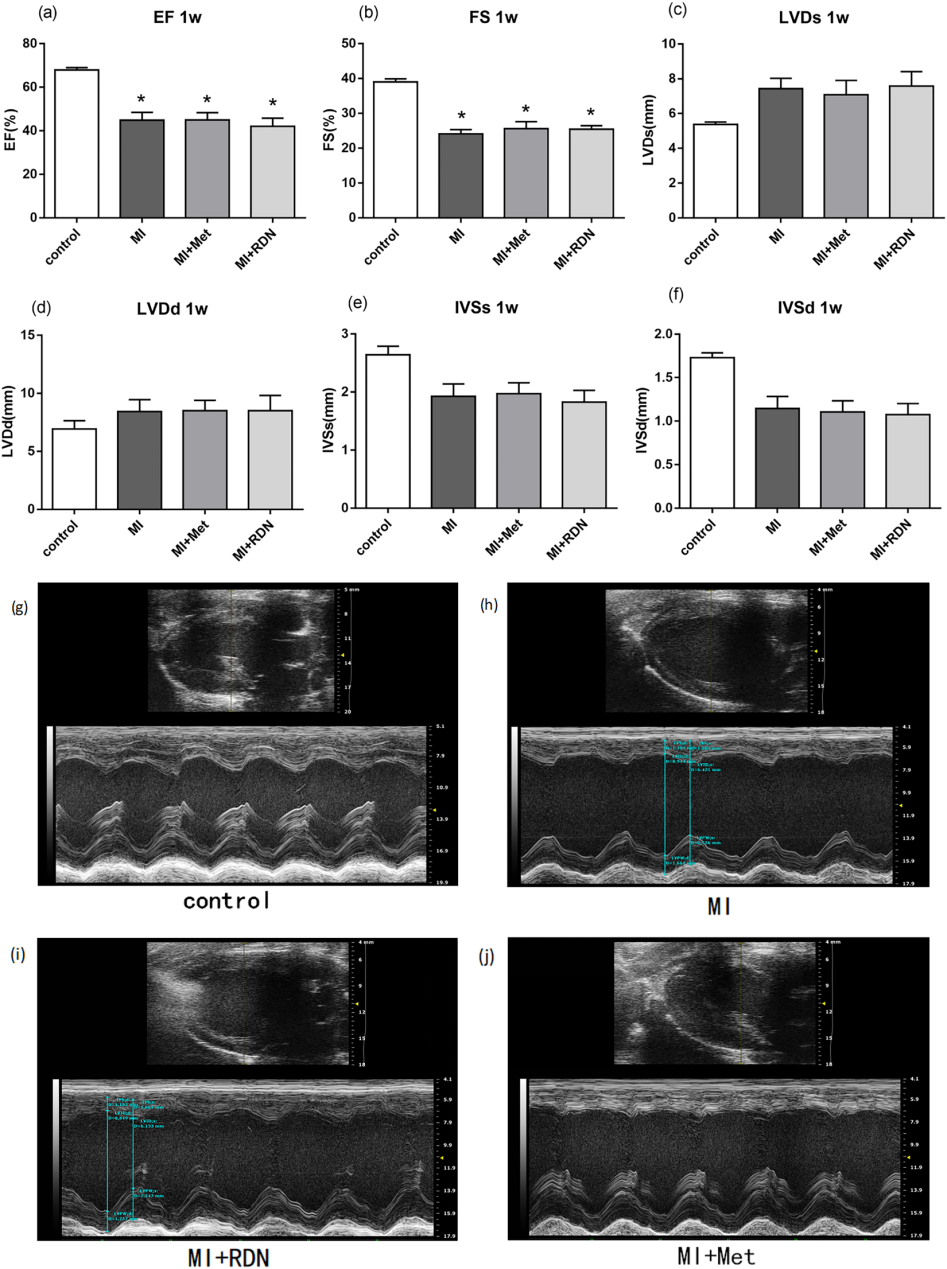
Cardiac function change at 1 week. Transthoracic echocardiography evaluation of (**a**) EF (**b**) FS (**c**) LVDs (**d**) LVDd (**e**) IVSs (**f)** IVSd. (**g**–**j**) Representative tracings of echocardiography in each group. (Data were mean ± SEM. *P < 0.05 vs. Control group). LVDs = left ventricular end systolic diameter; LVDd = left ventricular end diastolic diameter; EF = ejection fraction; FS = fractional shortening; IVSs = interventricular septal thickness in systole; IVSd = interventricular septal thickness in diastole.

At 1 week post-MI, echocardiography revealed that MI significantly decreased left ventricular ejection fraction (LVEF MI 44.77 ± 3.66% vs. Control 67.80 ± 1.14%, P = 0.0012) and left ventricular fractional shortening (LVFS MI 24.08 ± 1.24% vs. Control 38.96 ± 0.92%, P < 0.0001) compared with control group (Fig. 1). These indicated MI-induced ischemic cardiomyopathy model had been established.

### Cardiac function at 5 weeks

**Figure 2:**
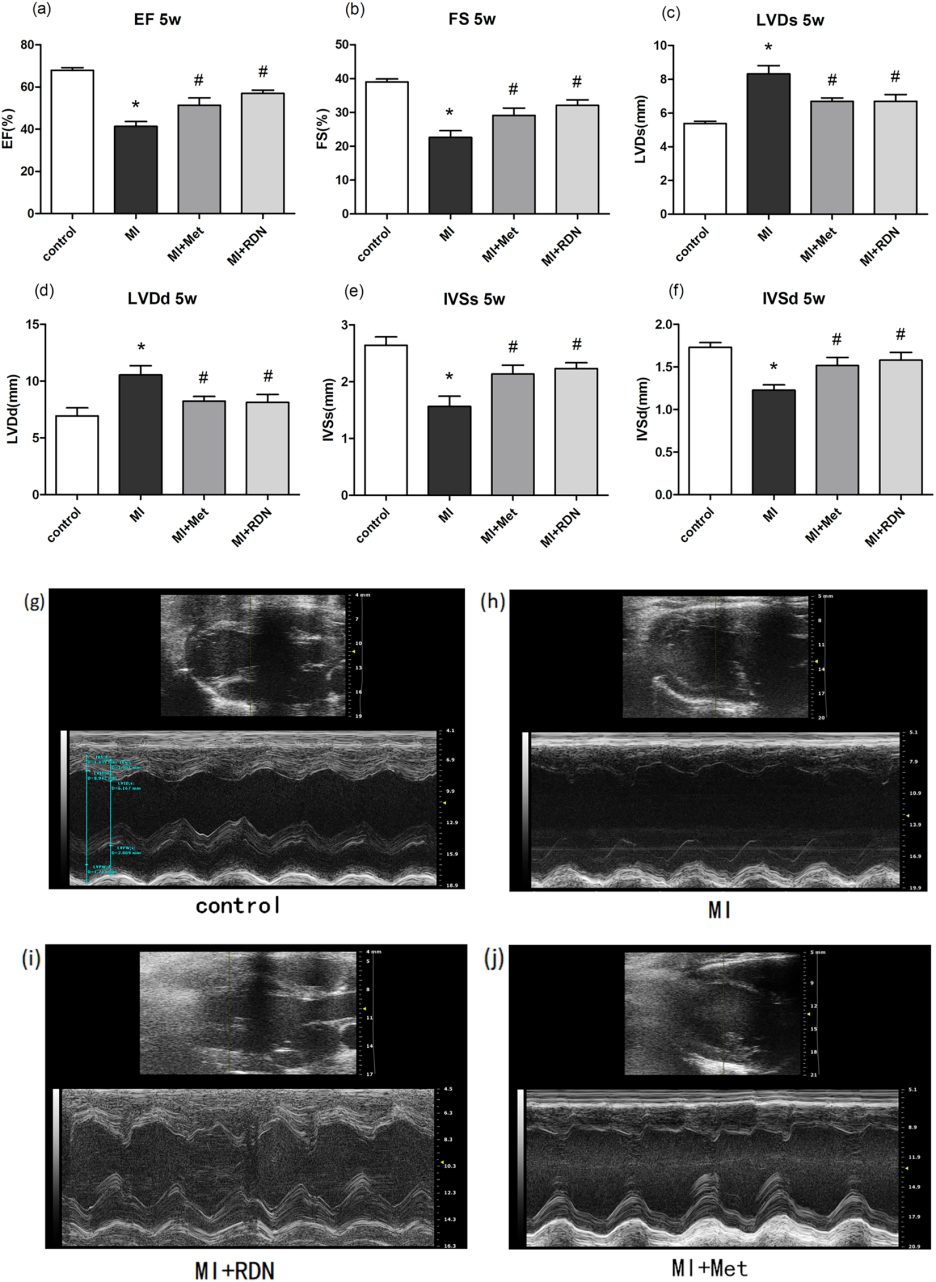
RDN and metoprolol partly restored cardiac function at 5 weeks. Transthoracic echocardiography evaluation of (**a**) EF (**b**) FS (**c**) LVDs (**d**) LVDd (**e**) IVSs (**f**) IVSd. (**g**–**j**) Representative tracings of echocardiography in control group, MI group, RDN group and Metoprolol group. (Data were mean ± SEM. *P < 0.05 vs. Control group; ^&^P < 0.05 vs. Met group). LVDs = left ventricular end systolic diameter; LVDd = left ventricular end diastolic diameter; EF = ejection fraction; FS = fractional shortening; IVSs = interventricular septal thickness in systole; IVSd = interventricular septal thickness in diastole.

At 5 weeks post-MI, there were 6, 8, 12 and 14 rats survived in control, MI, Met and RDN groups respectively. Compared with MI group, RDN and metoprolol significantly increased LVEF (RDN 56.99 ± 1.50% vs. MI, P < 0.0001; Met 51.36 ± 3.49% vs. MI, P = 0.0468; MI 41.34 ± 2.31%) and LVFS (RDN 32.08 ± 1.65% vs. MI, P = 0.0019; Met 29.10 ± 2.21% vs. MI, P = 0.0551; MI 22.62 ± 1.98% Fig. 2a,b). No significant difference was observed between RDN group and metoprolol group in LVEF (P = 0.1321) and LVFS (P = 0.2815). Furthermore, both RDN and metoprolol significantly decreased left ventricular end diastolic dimension (LVDd RDN 8.12 ± 0.71 mm vs. MI, P < 0.05; Met 8.23 ± 0.42 mm vs. MI, P < 0.05; MI 10.54 ± 0.82 mm) and left ventricular end systolic diameter (LVDs RDN 6.70 ± 0.39 mm vs. MI, P < 0.05; Met 6.69 ± 0.20 mm vs. MI, P < 0.05; MI 8.32 ± 0.49 mm) in comparison with MI group (Fig. 2c,d).

### Effectiveness of RDN

**Figure 3:**
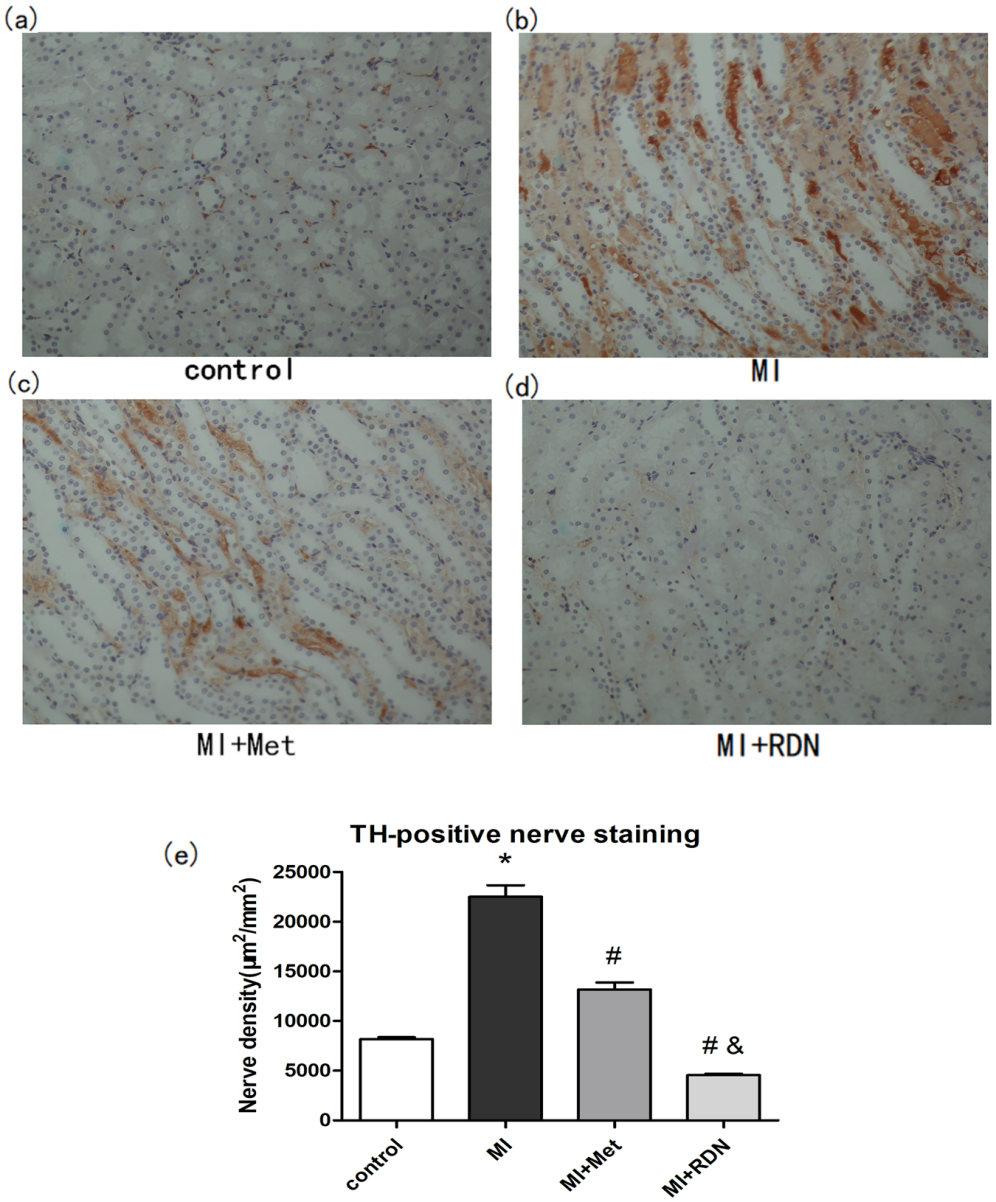
RDN significantly reduced renal expression of TH. (**a**–**d**) Representative images of immunohistochemical staining of renal TH protein expression in the control group, MI group, Met group and RDN group (magnification ×200). (**e**) Quantitative analysis suggested that TH expression in RDN group was significantly lower than that in MI group and Met group. (Data were mean ± SEM. *P < 0.05 vs. Control group; ^#^P < 0.05 vs. MI group; ^&^P < 0.05 vs. Met group).

At the end of the study period, Renal tyrosine hydroxylase (TH) staining was analyzed for the effectiveness of RDN. Compared with MI group, both RDN and metoprolol significantly reduced the densities of TH positive regions (RDN vs. MI, P < 0.01; Met vs. MI, P < 0.01). Besides, RDN significantly decreased the level of renal TH expression than metoprolol (Fig. 3). These confirmed the effectiveness of RDN.

### Effects of RDN on incidence of VAs

**Figure 4:**
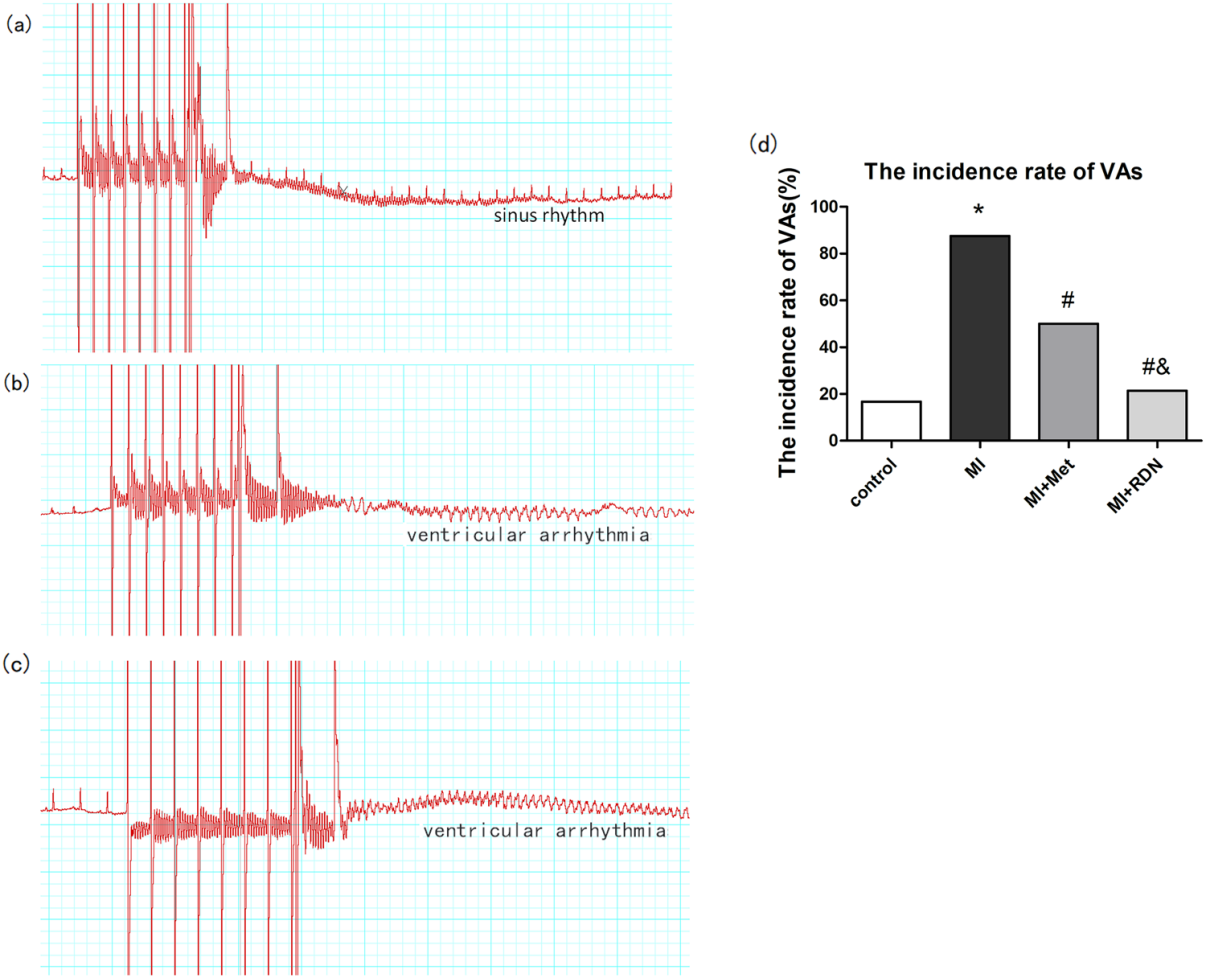
RDN significantly reduced the incidence of ventricular arrhythmias. Representative ECG of electrical stimulation, including sinus rhythm (**a**), ventricular arrhythmias (**b**) and (**c**). Ventricular arrhythmias were less easily induced in RDN group rather than in MI group and Met group (**d**). (*P < 0.05 vs. Control group; ^#^P < 0.05 vs. MI group; ^&^P < 0.05 vs. Met group).

At 5 weeks post-MI, electrical programmed stimulation (EPS) was performed in all groups. Compared with MI group, RDN and metoprolol significantly decreased the VA inducibility (RDN 3/14 vs. MI 7/8, P < 0.05; Met 6/12 vs. MI 7/8, P < 0.05; Control 1/6). Furthermore, the incidence of VAs was significantly decreased in RDN than Metoprolol group (RDN 3/14 vs. Met 6/12, P < 0.05) (Fig. 4).

### Effects of RDN on fibrosis in ventricle

**Figure 5:**
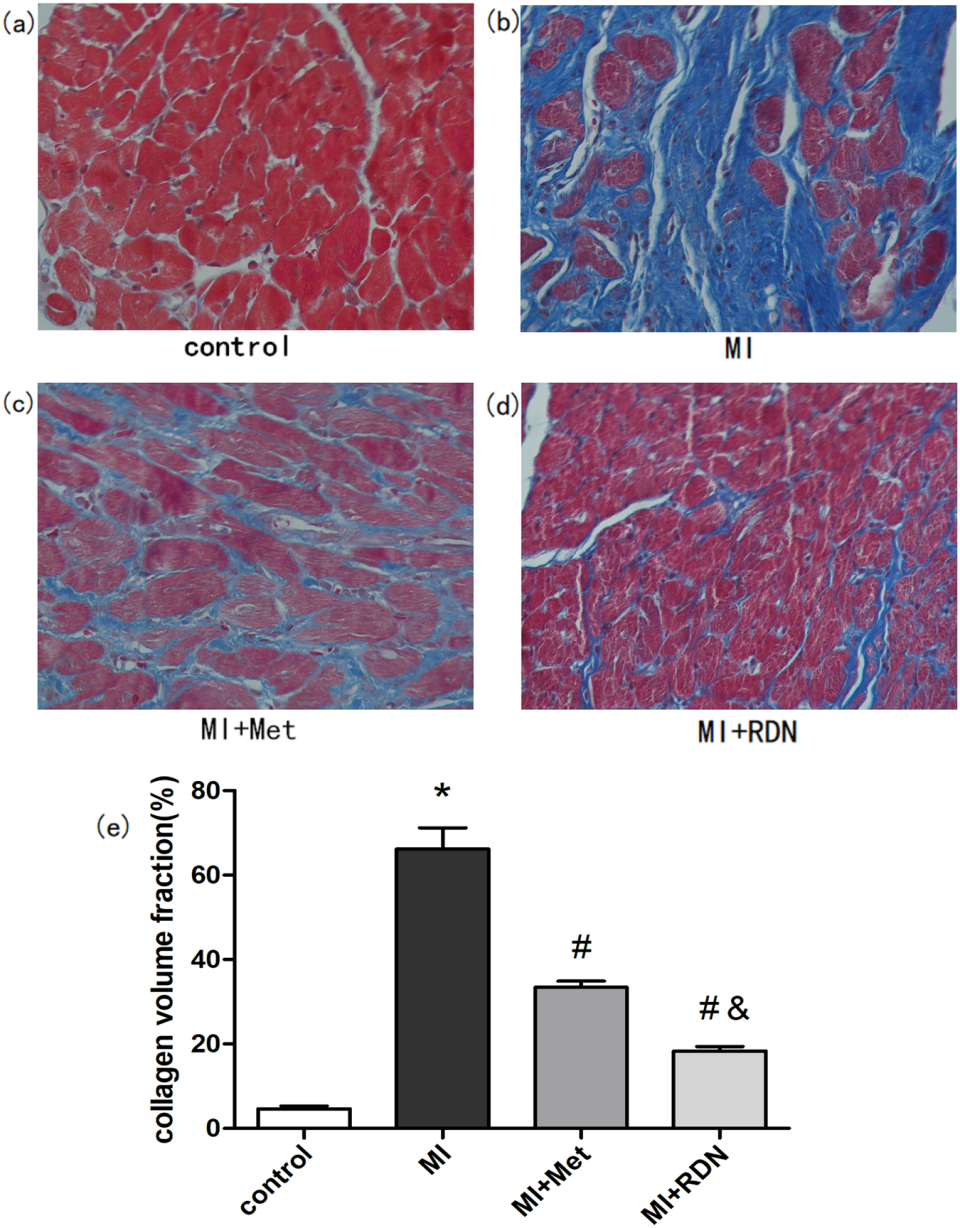
Both RDN and metoprolol significantly attenuated cardiac fibrosis. (**a**–**d**) Representative ventricular fibrosis (in blue) by Masson’s staining of samples from the control group, MI group, Met group and RDN group. (**e**) Quantitative analysis suggested that CVF in RDN group was significantly lower than that in MI group and Met group. (Data were mean ± SEM. *P < 0.05 vs. Control group; ^#^P < 0.05 vs. MI group; ^&^P < 0.05 vs. Met group).

Masson staining showed that both RDN and metoprolol group significantly reduced the collagen volume fraction (CVF) of left ventricle compared with the MI group (RDN 18.35 ± 1.06% vs. MI 66.21 ± 5.01%, P < 0.0001; Met 33.42 ± 1.47% vs. MI 66.21 ± 5.01%, P < 0.0001). Besides, RDN remarkably decreased CVF than metoprolol (RDN vs. Met, P < 0.0001, Fig. 5).

### Effects of RDN on Cx43 and p-Cx43

**Figure 6:**
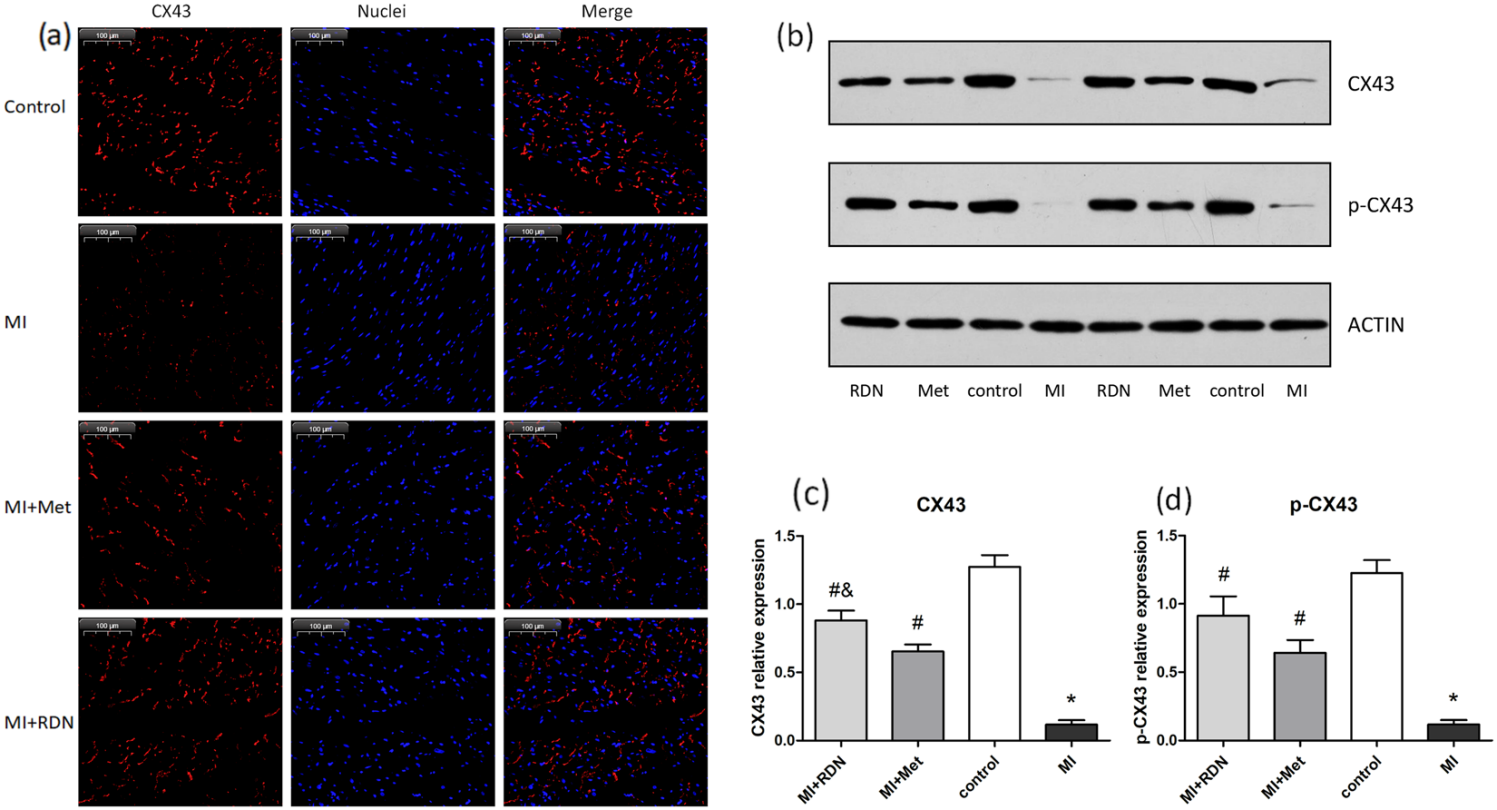
RDN and metoprolol significantly improved distribution and expression of Cx43 and p-Cx43 in the infarcted border zone compared with MI group. The distribution of Cx43 was disrupted in the border-zone of the infarcted area and the chaos of Cx43 was attenuated by RDN and metoprolol. (**b**) Representative cropped Western blot of Cx43, p-CX43 and ACTIN in heart. (**c**,**d**) Quantitative analysis of Cx43 and p-CX43 in heart by Western blot (Data were mean ± SEM. *P < 0.05 vs. Control group; ^#^P < 0.05 vs. MI group; ^&^P < 0.05 vs. Met group).

The localization of connexin was evaluated by immunofluorescence technique. Both RDN and metoprolol could significantly attenuate the chaos of connexin43 (Cx43), while metoprolol was less effective than RDN (Fig. 6a). The protein level of Cx43 and phosphorylated connexin43 (p-Cx43) were examined through western blotting. Compared with the MI group, both RDN and metoprolol significantly increased the relative expression of Cx43 (RDN vs. MI, P < 0.0001; Met vs. MI, P = 0.0001) and p-Cx43 (RDN vs. MI, P = 0.0015; Met vs. MI, P = 0.0018), Moreover, RDN is more effective than metoprolol in the expression of Cx43 (P = 0.039) but not p-Cx43 (P = 0.1577, Fig. 6).

### Effects of RDN on sympathetic neural remodeling

**Figure 7:**
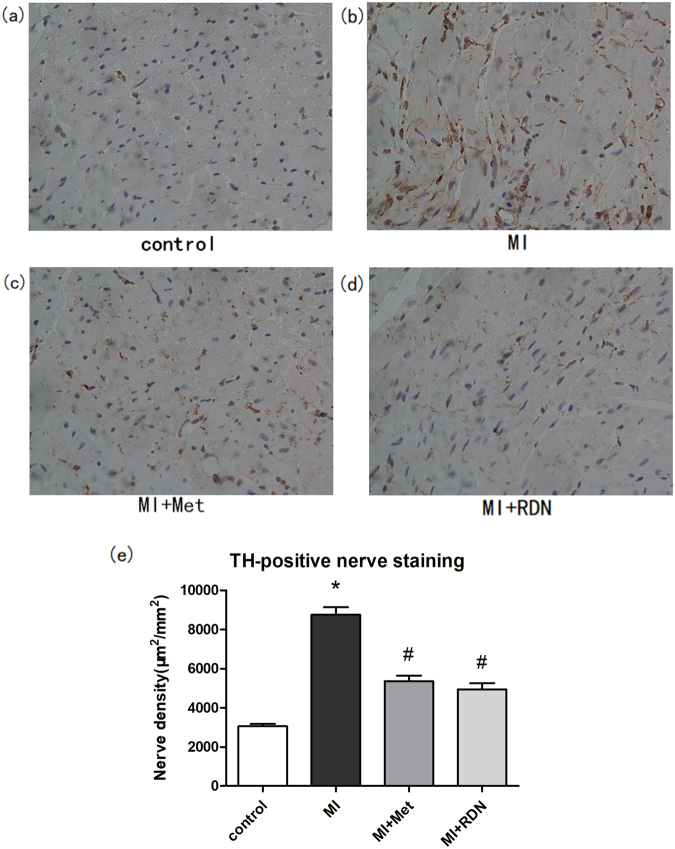
Both RDN and metoprolol significantly reduced cardiac expression of TH. (**a**–**d**) Representative images of immunohistochemical staining of cardiac TH protein expression in the control group, MI group, Met group and RDN group (magnification ×400). (**e**) Quantitative analysis suggested that TH expression in RDN group and metoprolol group were significantly lower than that in MI group. (Data were mean ± SEM. *P < 0.05 vs. Control group; ^#^P < 0.05 vs. MI group).

**Figure 8:**
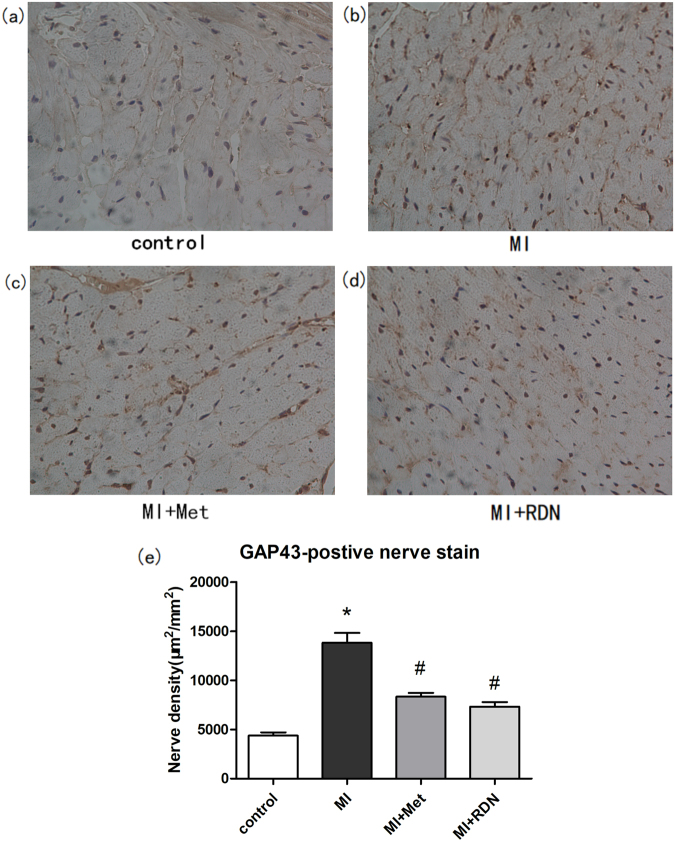
Both RDN and metoprolol significantly reduced cardiac expression of GAP. (**a**–**d**) Representative images of immunohistochemical staining of cardiac GAP protein expression in the control group, MI group, Met group and RDN group (magnification ×400). (**e**) Quantitative analysis suggested that GAP expression in RDN group and metoprolol group was significantly lower than that in MI group. (Data were mean ± SEM. *P < 0.05 vs. Control group; ^#^P < 0.05 vs. MI group).

The sympathetic activity in infarcted border zone (IBZ) was evaluated by immunostaining technique. Compared with the control group, MI significantly increased the densities of TH- (MI vs. Control, P < 0.0001) and growth associated protein 43 (GAP43) - (MI vs. Control, P < 0.0001) positive regions. Both RDN and metoprolol could significantly decrease the densities of TH- (RDN vs. MI, P < 0.0001; Met vs. MI, P < 0.0001) and GAP43- (RDN vs. MI, P < 0.0001; Met vs. MI, P < 0.0001) positive regions in comparison with MI group. However, no significant difference was observed between RDN and metoprolol in reducing densities of TH- (RDN vs. Met, P = 0.3532) and GAP43- (RDN vs. Met, P = 0.1221) positive regions (Figs 7 and 8).

## Discussion

The main findings of this study are as follows: (1) RDN and metoprolol significantly reduced VAs inducibility; (2) RDN and metoprolol decreased myocardial fibrosis; (3) RDN and metoprolol improved Cx43 expression and distribution, as well as sympathetic neural remodeling. RDN effects were not inferior to metoprolol in this ischemic cardiomyopathy model.

Myocardial fibrosis is an important substrate for reentry VAs genesis. In fact, many clinical studies showed that the level of fibrosis is an independent predictive factor for VAs both in ischemic and non-ischemic cardiomyopathy^22,23^. Our previous study demonstrated that RDN reduced electrical storm in cardiomyopathy patient with ICD, which may be associated with fibrosis^24^. In patients with Brugada syndrome, VAs are associated to epicardial fibrosis in right ventricular outflow tract, and catheter ablation in this fibrosis area could significantly reduce VAs events^25^. Fibrosis is a critical pathway in organ injury^26^. In response to MI, structural remodeling happens in the infarcted regions, with massive deposition of extracellular matrix. After that, cytokines such as TGF-β1 are released and fibroblasts are activated, which could ultimately develop into myocardial scar and cardiac fibrosis^26^. Myocardial fibrosis is an important substrate for VAs genesis^27^. Firstly, fibrous tissue could develop into regions of conduction block and form nonuniform anisotropy and slow conduction, which may finally results in reentry substrate for sustained VAs^28^. Secondly, the abnormal myocytes in regions of interstitial fibrosis might develop spontaneous diastolic depolarization, leading to abnormal automaticity^29^. Furthermore, fibroblasts also play a direct role in modulating the electrophysiology by acting as obstacles and depolarizing myocytes. Thus, when the fibroblast further increases, the electrical conduction slows down and finally leads to conduction failure^27^. In our previous studies, we had demonstrated that RDN could significantly attenuate organ fibrosis. In this study, we found that RDN is superior to metoprolol in reducing cardiac fibrosis^12,30^, which may explain the lower incidence of VAs.

Cx43 is the predominant ventricular gap junction protein with the function of rapidly spreading and coordinating excitation signals for an effective heart contraction^31,32^. Thus, it is critical for maintaining normal cardiac electrical conduction^33^. After MI, the de-phosphorylation of Cx43, the reduced expression and the disarrayed distribution of Cx43 act synergistically on conduction abnormalities and reentrant arrhythmias^34–36^. Firstly, the expression of Cx43 influences the susceptibility to VAs^37^ after MI and the down-regulation of CX43 alter the spread of the impulse of ventricular myocardium. Roell *et al*.^38^ reported that engraftment of connexin43-expressing cells can prevent post-infarct arrhythmia. On the contrary, Lerner *et al*.^39^ demonstrated that in Cx43-deficient mice, the incidence of VAs increased markedly when coronary artery occluded. Secondly, abnormal distribution of Cx43 in infarct-related myocardium can cause abnormal conduction. After MI, Cx43 arrayed longitudinally along the lateral surfaces of the myocardial cells while normally distributed in intercalated disc^40^. Those changes not only reduce electrical conductance, but also decrease permeability of chemicals between cardiomyocytes^41^. Thus, changes of gap junction protein are the basis of arrhythmia after MI^42^. According to our study, we confirmed that RDN can increase the expression of Cx43 in the IBZ and ameliorate the distribution of Cx43 in the intercalated disc, which led to better coordination and synchronization of electrical activity and restored conduction and contractile function, Thus, RDN can effectively inhibit formation of arrhythmias. The level of Cx43 in the metoprolol-treated group was also partially ameliorated, but inferior to RDN group.

Sympathetic neural remodeling also account for the occurrence of arrhythmias after MI. Firstly, it has been widely recognized that MI could result in degeneration and death of sympathetic fibers in both infarcted area^43^ and the viable myocardium^44^. Due to excessive express of nerve growth factor (NGF)^45^, the degenerative sympathetic nerve could ultimately develop into sympathetic neural remodeling which manifests as cardiac nerve sprouting and sympathetic hyperinnervation^46^. Secondly, it has been demonstrated that excessive sympathetic activity in heart can directly lead to arrhythmias in both post-MI patients and animal models^47^. The potential mechanisms may be as follows: On one hand, the heterogeneous of sympathetic transmission after MI contributes to a nonuniform electrophysiologic response, which could create a substrate for VAs^48^. On the other hand, symptomatic hyperinnervation can also lead to lower ventricular tachycardia (VT) and ventricular fibrillation (VF) threshold, thus contributing to the occurrence of arrhythmias by altering the electrophysiological properties of the innervated myocardium^49^. In addition, Gu *et al*.^50^ also demonstrated that increased sympathetic tone promoted the degradation and de-phosphorylation of Cx43, which may work together and contribute to VAs. The present study found that both RDN and metoprolol can significantly inhibit the densities of TH and GAP43 positive nerve fibers in IBZ, indicating that RDN and metoprolol can effectively inhibit sympathetic neural remodeling in post-MI rats. This may be another potential mechanism to reduce arrhythmias.

Nowadays, the potential of RDN for arrhythmias and the mechanistic evaluation is ongoing. Jackson *et al*.^51^ reported that RDN reduces spontaneous VAs in the subacute period after acute MI by inhibiting sympathetic neural remodeling. They demonstrated that in a relatively small infarct size (mean LVEF at 55%), abnormal automaticity in response of increased cardiac sympathetic activity might be the major mechanism. In our study, where infarct size was big and sustained VAs were induced by EPS, the distribution of Cx43 and the formation of scarring may have been another prominent mechanism. After a wide range of infarct size, a large number of gap junction channels close and a region of electrical conduction block forms, leading to delayed arrhythmias^52^. Chang *et al*.^53^ showed that RDN could improve calcium dynamic and decrease susceptibility to arrhythmogenic cardiac alternans by inhibiting sympathetic nerve activity, thereby reducing the incidence of VAs after heart failure. In addition to the sympathetic activity mentioned by Chang *et al*., intracellular calcium handling is more relevant to cardiac contractile function. Previous study showed that HF destroyed calcium cycling in sarcoplasmic reticulum, further influenced amplitude and duration of calcium transients, resulting in calcium transient alternans and action potential duration alternans^54^. In the present study, we found that RDN can effectively improve cardiac function after MI and may further modify calcium regulation abnormalities and reduce the incidence of conduction block and VAs, which also supports the findings reported by Chang *et al*. In addition to these conventional mechanisms, the natriuretic peptide system has become a new target for cardiovascular events. Polhemus *et al*.^55^ found that RDN can protect the failing heart via inhibition of neprilysin activity. Diego *et al*.^56^ demonstrated that neprilysin inhibition decreased VAs in patients with reduced ejection fraction heart failure. Except renin-angiotensin pathway and adrenergic system, inhibition of neprilysin activity by RDN may be another possible mechanism for the reduction of VAs in rats after MI.

Previous study on the comparison of efficacy of RDN versus β-blocker on VAs was limited to the acute period of MI. Linz *et al*.^57^ demonstrated that the effect of RDN on VAs during acute ischemia period was similar to β-blockers. The occurrence of VAs on the early period might result from the triggered activity from Purkinje fibers in ischemic regions in response to increased catecholamine levels^58^. Hence, RDN might have similar effect with β-blockers on reducing the activity of catecholamine during the early period. But during the subacute period after MI, except the increased activity of catecholamine, VAs could also result from sympathetic neural remodeling, the distribution of Cx43^38^ and cardiac fibrosis. In conclusion, according to this study, RDN is not inferior to β-blocker in reducing the remodeling of connexin43 and sympathetic nerve after MI.

## Limitations

The current study existed several limitations. Firstly, RDN in this study was performed only on the adventitia of the renal artery, which differs from clinical renal artery ablation. Secondly, cardiac dysfunction is one of the predictors of VAs in ischemic cardiomyopathy. In this study, RDN can improve the cardiac function after MI. However, we did not investigate the relationship between cardiac function and VAs. Hence, whether the reduction of VAs benefits from improvements in cardiac function requires further study. Furthermore, despite reported by relevant literature^55,56^, we did not further confirm the direct effect of RDN on VAs by inhibiting neprilysin.

## Conclusions

RDN is effective on reducing VAs inducibility and the effect is not inferior to Metoprolol. The mechanism could be associated with cardiac fibrosis reducing, Cx43 expression regulating and sympathetic nerve remodeling.

## Materials and Methods

### Animals and Experimental Protocols

All procedures in this study were approved by the Ethics Committee of Nanjing Medical University. The animal experiments were performed conform the Guide for the Care and Use of Laboratory Animals (National Institutes of Health publication 8th edition, 2011). Sixty six-week male Sprague-Dawley rats (200–220 g) were provided by Nanjing Medical University Laboratory Animal Center. After a one-week-adaption, 54 rats underwent ligation of left anterior descending coronary artery to induce MI, while 6 rats underwent sham-MI surgery served as the control. Subsequently, 47 surviving MI rats were randomly divided into 3 groups by random number method: MI with RDN operation (RDN group, n = 16), MI with metoprolol (metoprolol group, n = 16) and MI with sham-RDN operation (MI group, n = 15). Metoprolol was administrated intragastrically at a dose of 20 mg/kg/day for 5 weeks starting on day 1 after MI surgery^59,60^. The dosing regimen of metoprolol was based on previous study^61^. The RDN or Sham-RDN procedure was performed bilaterally 1 week after MI surgery. All the data in this research was collected by double-blind experiment.

### Myocardial infarction model

MI model was created as described previously. In brief, the rats were weighted before intraperitoneal injection with 2% sodium pentobarbital (50 mg/kg). After they were anesthetized, the rats were endotracheally intubated and mechanically ventilated. Thoracotomy was performed at the forth intercostal space, then the left anterior descending coronary artery was ligated with a 7–0 silk suture at about 1–2 mm at the starting point of the branch. After ligation, a local pale could be seen at the surface of heart, and then sutured the thoracic cavity and skin. All the rats underwent myocardial infarction were given penicillin intramuscularly in order to prevent infection. Rats in control group only underwent thoracotomy without ligation. All MI procedures were performed by the same operators.

### Renal denervation model

One week after MI surgery, rats in RDN group underwent the second operation. RDN was implemented as described previously. The rats were anesthetized by intraperitoneal injection with 2% sodium pentobarbital (50 mg/kg). Next, the skin was cut at about 2 transverse fingers below the costalspinal angle in order to find the kidneys and perirenal adipose tissue. And then, all visible nerves were severed. After that, 20% phenol in an alcohol solution were painted on renal vessels to destroy the remaining nerves. Same procedures were performed on the rats in sham group just without the destruction of the nerves. The RDN/sham-RDN procedure was conducted by the same operators.

### Electrical Programmed Stimulation

Five weeks post MI, rats underwent ventricular electrical programmed stimulation before sacrificed. After anesthetized by intraperitoneal injection with 2% sodium pentobarbital (50 mg/kg), the rat was recorded electrocardiography by three needle electrodes placed on right upper limb and legs. And then, the EPS was used to stimulate the left ventricular apex of the heart through a bipolar electrode and the incidence of VAs was investigated. By a cycle length of 140 ms, the threshold potential for stable pacing was gained. Pacing was started with twice as much as the threshold and the cycle length of 140 ms, which was the interval of 8 stimulus (S1). An extra stimulus (S2) was applied until failed to induce ventricular depolarization, while the interval between S1 and S2 was progressively shortened by 10 ms. The operators were blinded to the treatment of the rats.

### Echocardiography

Echocardiography was performed at week 1 and week 5 before RDN and EPS, respectively. After the rats were anesthetized with isoflurane, the structure and function were evaluated by Vevo2100 (VisualSonics, Canada) system equipped with a MS-250, 16.0–21.0 MHZ imaging transducer. The investigators were blinded to the treatment of the rats.

### Histological analysis

Rats were sacrificed immediately after EPS. The heart and bilateral kidneys were taken after perfusion with phosphate-buffered saline and washed with phosphate buffered saline. The heart was cut horizontally along the long axis at the pale area of infarct and fixed with 4% paraformaldehyde for 24 hours. The longitudinal kidney was also cut along the long axis incision and fixed with 4% paraformaldehyde for 24 hours. After being fixed, dehydration, and paraffin embedding, the heart and kidney were made into pathological sections. Hearts were subjected to Masson’s trichrome staining, TH staining and GAP43 staining. Kidneys were subjected to tyrosine hydroxylase staining. After staining, they were observed under a normal light microscope and six representative fields were randomly selected for analysis by Image-Pro Plus 6.0.

### Immunofluorescence labelling

Immunofluorescence labelling was used to investigate the distribution of Cx43 in the infarcted border zone. The samples were evaluated under a fluorescence microscope (Nikon, Japan).

### Western Blot

Protein expression of Cx43 (Abnova, China) and p-Cx43 (Abnova, China) in myocardial tissue were detected by western blotting technique. After the heart samples were lysed in lysis buffer (Abnova, China), the protein concentrations were determined using the BCA method as previously. ACTIN (Abnova, China) was used to normalize protein levels.

### Statistical analysis

SPSS 16.0 software was used for statistical analysis, and GraphPad Prism5 software was for mapping. Quantitative data were shown as mean ± SEM, for two-group comparison, data were analyzed with two-tailed unpaired t tests, for multiple-groups comparisons, data were performed using one-way ANOVA followed by LSD test. Qualitative data were analyzed with Fisher exact test. P < 0.05 was considered statistically significant.

### Data availability

The datasets generated and analyzed during the current study are available from the corresponding author on reasonable request.
